# Early tinnitus burden and subjective hearing are candidate markers of 2-year quality of life after cochlear implantation in single-sided deafness

**DOI:** 10.3389/fnins.2026.1832641

**Published:** 2026-04-15

**Authors:** Jasper Karl Friedrich Schrader, Moritz Gröschel, Agnieszka J. Szczepek, Heidi Olze

**Affiliations:** 1 Department of Otorhinolaryngology, Head and Neck Surgery, Charité – Universitätsmedizin Berlin, Berlin, Germany; 2 Faculty of Medicine and Health Sciences, University of Zielona Góra, Zielona Góra, Poland

**Keywords:** candidate markers, cochlear implant, Nijmegen cochlear implant questionnaire, perceived stress, postoperative follow-up, quality of life, single-sided deafness, tinnitus

## Abstract

**Background:**

Cochlear implantation is a common treatment for adults with single-sided deafness (SSD), but patient-reported benefits vary. The relationships among tinnitus burden, perceived hearing ability, psychological distress, disease-specific health-related quality of life, and whether early postoperative outcomes predict later results are not well understood.

**Objective:**

This study explores how disease-specific quality of life relates to tinnitus burden, hearing, stress, depression, and anxiety after cochlear implantation in SSD. It also seeks early markers linked to 2-year outcomes.

**Methods:**

This secondary complete-case analysis was based on a previously reported prospective longitudinal SSD cohort. Of 70 adults with postlingual SSD, 36 (51.4%) had complete Nijmegen Cochlear Implant Questionnaire (NCIQ) data at baseline and at 6 months, 1 year, and 2 years after unilateral cochlear implantation and were included. Additional measures included the Tinnitus Questionnaire (TQ), Oldenburg Inventory (OI), PerceivFed Stress Questionnaire (PSQ), General Depression Scale (ADS-L), Generalized Anxiety Disorder 7-item scale (GAD-7), and Freiburg Monosyllable Test (FMT) at 65 dB. Timepoint-specific correlations with the NCIQ were analyzed using Spearman’s rank correlations. Exploratory multivariable analyses employed linear regression on rank-transformed variables to assess whether baseline and 6-month patient-reported profiles were associated with 2-year NCIQ outcomes. Longitudinal within-patient comparisons were conducted as a secondary descriptive analysis.

**Results:**

Higher NCIQ scores were linked to lower tinnitus burden and better hearing across all assessments. Associations with depression and anxiety persisted, while connections with perceived stress emerged after surgery. At baseline, higher tinnitus burden was associated with lower 2-year NCIQ scores. At 6 months, higher tinnitus is still associated with lower 2-year NCIQ scores, whereas better hearing is associated with higher 2-year NCIQ scores. Early postoperative improvement was followed by stabilization over 2 years.

**Conclusion:**

Improvement in health-related quality of life after cochlear implantation in adults with SSD is complex and extends beyond hearing alone. Tinnitus was the most consistent negative factor, while improved subjective hearing at 6 months was associated with better outcomes at 2 years. These results support a structured, multidimensional approach to patient-reported follow-up after cochlear implantation in SSD and suggest that early postoperative patient-reported status may serve as an early candidate marker for later quality-of-life outcomes.

## 1 Introduction

Single-sided deafness (SSD) is a distinct form of sensorineural hearing loss in which, despite normal hearing in the contralateral ear, affected individuals often experience substantial functional and psychosocial burden, including impaired sound localization, reduced speech understanding in noisy environments, tinnitus, and increased stress, anxiety, and depression (Van de Heyning et al., 2016; Bassiouni et al., 2023; Häußler et al., 2020; Arndt et al., 2011; Daher et al., 2023; Oh et al., 2023; Vanderauwera et al., 2020; Wesarg et al., 2024; Wendrich et al., 2024).

In recent years, cochlear implantation has emerged as an effective treatment option for SSD and has often been shown to outperform alternative approaches such as bone-conduction devices or contralateral routing of signals (CROS), partly by restoring bilateral auditory input and thereby improving spatial hearing and speech understanding in complex listening environments (Arndt et al., 2011; Daher et al., 2023; Oh et al., 2023; Wesarg et al., 2024; Wendrich et al., 2024; Marx et al., 2021). These benefits are especially relevant in SSD because, unlike in bilateral deafness or asymmetric hearing loss, treatment goals often extend beyond restoration of basic communication to include relief from tinnitus, improved hearing performance in noise, and reduction of psychosocial burden. Accordingly, patient-reported outcome measures may be particularly important for capturing treatment benefit in this group (Van de Heyning et al., 2016; Bassiouni et al., 2023; Arndt et al., 2011; Olze et al., 2012; Bekele Okuba et al., 2023).

Previous work from the parent cohort on which the present study is based demonstrated significant longitudinal improvements over 2 years in health-related quality of life, subjective hearing, tinnitus burden, and selected psychological outcomes after cochlear implantation in SSD (Schrader et al., 2026). The aim of the present manuscript is not to re-report those longitudinal outcome data, but to address a complementary and previously unanswered question within the same prospective cohort: which patient-reported domains are most closely linked to disease-specific health-related quality of life at different postoperative stages, and whether early patient-reported outcome measure profiles are associated with later 2-year outcomes on the Nijmegen Cochlear Implant Questionnaire (NCIQ). This distinction is clinically relevant because patients with SSD may show similar average postoperative improvement at the group level while differing substantially in the pattern and durability of perceived benefit. Current evidence on factors associated with patient-reported outcomes in SSD remains limited and heterogeneous, particularly regarding tinnitus-related burden, subjective hearing experience, and broader psychosocial measures. The present secondary analysis was therefore designed to extend, rather than duplicate, the parent-cohort report by focusing on correlates and candidate early markers of later disease-specific quality-of-life outcomes.

Therefore, this study was conducted as a secondary complete-case analysis with two main goals: first, to examine the relationship between the Nijmegen Cochlear Implant Questionnaire (NCIQ) and the Tinnitus Questionnaire (TQ), Oldenburg Inventory (OI), Perceived Stress Questionnaire (PSQ), General Depression Scale (ADS-L), and Generalized Anxiety Disorder 7-item scale (GAD-7) at specific time points (baseline, 6 months, 1 year, and 2 years) after cochlear implantation; and second, to examine whether baseline and early postoperative status were associated with the 2-year NCIQ score. As a secondary descriptive objective, we also characterized longitudinal postoperative changes in the complete-case subset to confirm that its overall trajectory was similar to that previously observed in the parent cohort.

## 2 Materials and methods

### 2.1 Participants

This study was a secondary complete-case analysis of a previously described prospective longitudinal cohort of 70 adults with postlingual SSD who underwent unilateral cochlear implantation at a specialized tertiary referral center in Berlin, Germany (Schrader et al., 2026). Of these 70 patients, 36 (51.4%) had complete Nijmegen Cochlear Implant Questionnaire (NCIQ) data at baseline, 6 months, 1 year, and 2 years and therefore constituted the analytic cohort for the present study, whereas 34 patients (48.6%) were not included because at least one required NCIQ assessment was missing. This study was conceived as a secondary complete-case analysis addressing a distinct analytic objective from the previously published parent-cohort report. While the parent-cohort publication focused on longitudinal postoperative changes in the entire SSD sample, this analysis examined two related questions within the subset that had complete 2-year NCIQ follow-up: first, whether NCIQ total scores were linked to tinnitus burden, subjective hearing ability, perceived stress, depressive symptoms, and anxiety at each assessment point; and second, whether baseline or early postoperative patient-reported profiles related to 2-year NCIQ outcomes. Longitudinal pairwise comparisons were retained only as a secondary descriptive analysis to characterize the analytic subset and to verify that its overall postoperative trajectory was broadly consistent with that previously reported for the full cohort.

Adults with postlingual SSD who received unilateral cochlear implants between 2013 and 2021 were screened for inclusion. SSD was defined as a pure-tone average across 0.5, 1, 2, and 4 kHz of at least 70 dB HL in the poorer ear and 30 dB HL or less in the better ear. Additional audiological eligibility criteria included aided speech recognition of 60% or less in the poorer ear on the Freiburg monosyllable test at 65 dB SPL and good speech recognition in the better-hearing ear. For this analysis, participants were included only if complete NCIQ data were available at baseline, as well as at 6 months, 1 year, and 2 years after implantation. To assess potential selection biases stemming from the complete-case design, baseline demographic and clinical characteristics of patients included and excluded from the parent cohort were compared. Detailed reasons for each missed follow-up assessment were not consistently available in this long-term real-world cohort.

### 2.2 Ethics

The study was approved by the Ethics Committee of Charité – Universitätsmedizin Berlin (EA2/030/13; first approval 11 March 2013; amendment 30 March 2017). All participants provided written informed consent prior to enrollment and participation.

### 2.3 Inclusion criteria

Eligible participants were those who met these criteria: age of 18 years or older at the time of implantation; postlingual single-sided deafness (SSD), characterized by severe-to-profound sensorineural hearing loss in the poorer ear (pure-tone average, PTA, of 70 dB HL or more across 0.5, 1, 2, and 4 kHz) and normal to mild hearing loss in the other ear (air-conduction thresholds of 30 dB HL or less); speech understanding of 60% or less in the poorer ear with hearing aids, assessed with the Freiburg monosyllable test at 65 dB SPL, in line with the German S2k guideline; good speech understanding in the better-hearing ear (80% or more in the Freiburg monosyllable test at 70 dB SPL); planning for unilateral cochlear implantation at the study site; ability and willingness to complete Patient-Reported Outcome Measures (PROMs); and providing written informed consent.

### 2.4 Exclusion criteria

Participants were excluded if any of the following conditions applied: age under 18; prelingual deafness or congenital unilateral hearing issues; follow-up less than 6 months or inadequate postoperative PROM data; language barriers preventing valid questionnaire completion; neurological, cognitive, or psychiatric conditions that could impair participation or PROM accuracy; previous cochlear implants or other active middle- or inner-ear procedures on the poorer-hearing ear; active middle-ear issues contraindicating cochlear implantation during evaluation; or hearing loss that does not meet SSD criteria.

### 2.5 Test battery and outcome measures

#### 2.5.1 Freiburg monosyllabic test (Freiburger Einsilbertest, FMT)

Speech intelligibility was evaluated using the Freiburg Monosyllable Test, a standardized German speech audiometry tool that measures word recognition performance with lists of phonetically balanced monosyllabic words presented at a consistent sound level (Hoth, 2016). Test results are reported as a percentage correct (%), with higher scores indicating better speech recognition. In this study, the Freiburg monosyllable test was conducted in a free-field setup with external loudspeakers positioned at 0° (facing the patient), delivering sound at a sound pressure level of 65 dB. During the test, the contralateral (better-hearing) ear was masked.

#### 2.5.2 Patient-reported outcome measures (PROMs)

##### 2.5.2.1 Nijmegen cochlear implant questionnaire (NCIQ)

The NCIQ is a tool designed to measure health-related quality of life in adult cochlear implant users (Hinderink et al., 2000). It contains 60 items, organized into three main domains (physical, psychological, and social functioning) and six subdomains: basic sound perception (NCIQ1), advanced sound perception (NCIQ2), speech production (NCIQ3), self-esteem (NCIQ4), activity limitation/activity (NCIQ5), and social interaction (NCIQ6). Items are answered on a five-point Likert scale and converted into subscale and total scores on a 0–100 scale, with higher scores indicating better outcomes.

##### 2.5.2.2 Oldenburg inventory (OI)

The OI is a self-report measure of everyday hearing abilities (Holube and Kollmeier, 1991; Holube and Kollmeier, 1994). In this study, the 12-item short version was used, covering three functional listening domains: listening in quiet, listening in noise, and directional hearing or sound localization. Items are rated on a 5-point scale (1–5) that reflects increasing difficulty or impairment, and scale scores are usually summarized as mean values for each domain and as a total score. OI scores range from 1 (poor subjective hearing ability) to 5 (excellent subjective hearing ability), with higher scores indicating better perceived hearing performance.

##### 2.5.2.3 Tinnitus questionnaire (TQ)

The German Tinnitus Questionnaire (TQ) is a commonly used tool in German-speaking countries to measure distress caused by tinnitus (Goebel and Hiller, 2003; Hiller and Goebel, 1992). It consists of 52 items that cover various areas, including emotional and cognitive distress, intrusiveness, sleep problems, physical complaints, and hearing difficulties. Responses are rated on a three-point scale, yielding a total score in which higher values indicate more severe tinnitus-related distress. In this study, the TQ total score was analyzed as a continuous measure of tinnitus-related distress.

##### 2.5.2.4 Perceived stress questionnaire (PSQ)

Perceived stress was assessed using the PSQ, which measures an individual’s current subjective experience of stress over the past 4 weeks (Fliege et al., 2001). The PSQ comprises four subscales—worries, tension, joy, and demands—each with 5 items. Scores are usually converted to a 0–1 scale, where higher scores indicate greater perceived stress (noting that the “joy” dimension is inversely related to stress in scoring conventions). As a practical interpretive guideline, scores between 0.45 and 0.60 indicate moderate stress perception, while scores above 0.60 indicate high stress perception.

##### 2.5.2.5 Depression measurement: general depression scale (Allgemeine Depressionsskala-Langform, ADS-L)

Depressive symptoms were measured using the ADS-L, a 20-item self-report questionnaire that assesses the frequency of typical depressive symptoms—including affective, cognitive, somatic, and social—over the past week (Hautzinger et al., 2012). Each item is rated on a 4-point scale, and the total score (usually ranging from 0 to 60) reflects symptom severity, with higher scores indicating more severe depressive symptomatology. A cutoff score of ≥23 is often used to identify clinically significant depressive symptoms in screening settings.

##### 2.5.2.6 General anxiety disorder 7-item scale (GAD-7)

Anxiety symptoms were assessed using the GAD-7, a 7-item tool that measures how often core anxiety symptoms occur over the past 2 weeks (Spitzer et al., 2006). Items are scored from 0 to 3 (not at all to nearly every day), yielding a total score of 0 to 21, with higher scores indicating more severe anxiety. Standard cut-points of 5, 10, and 15 define mild, moderate, and severe anxiety levels, respectively.

### 2.6 Statistical analysis

Statistical analysis was conducted using IBM SPSS Statistics (IBM-Corp, 2024). The Kolmogorov–Smirnov test indicated that most variables were not normally distributed. Accordingly, cross-sectional and longitudinal bivariate analyses were performed using nonparametric methods. For the multivariable analyses, linear regression on rank-transformed variables was used as an exploratory approach to examine multivariable monotonic associations while reducing sensitivity to non-normality and outliers in this modest-sized sample.

Because the primary goal of this secondary analysis was to examine timepoint-specific associations and early candidate variables associated with the 2-year health-related quality-of-life outcome, the main inferential analyses included cross-sectional correlation analyses and prospective rank-based regression models. Cross-sectional associations between health-related quality of life and auditory and psychological variables were analyzed separately at baseline, 6 months, 1 year, and 2 years after cochlear implantation. At each time point, the primary outcome variable was the total score of the Nijmegen Cochlear Implant Questionnaire (NCIQ total). Corresponding associated variables included Tinnitus Questionnaire total score (TQ total), Oldenburg Inventory total score (OI total), Perceived Stress Questionnaire total score (PSQ), General Depression Scale (ADS-L), and Generalized Anxiety Disorder 7-item scale score (GAD-7). Spearman’s rank-order correlation coefficients (rho) were calculated using two-tailed significance testing and pairwise handling of missing data.

To investigate whether early patient-reported status was linked to long-term health-related quality of life, two separate exploratory rank-based multiple regression models were estimated, with the 2-year NCIQ total score as the dependent variable. In the first model, the baseline variables TQ total, OI total, PSQ, ADS-L, and GAD-7 were entered simultaneously. In the second model, the corresponding 6-month variables were entered simultaneously. For each model, the dependent variable and all predictors were ranked, and standard multiple linear regression (enter method) was then performed on the ranked data. These analyses were based on listwise deletion of missing values. Model fit was assessed using *R*, *R*^2^, adjusted *R*^2^, and the overall *F* test, and variables were described using unstandardized regression coefficients (B), standardized coefficients (*β*), 95% confidence intervals, and *p* values. Because of the exploratory design, single-center setting, and modest sample size, these models were intended to identify candidate early markers and associated variables rather than to derive or validate an individual-level clinical prediction tool. Statistical significance was set at *p* < 0.05.

Longitudinal within-patient comparisons were included as a secondary descriptive analysis to characterize the subset and verify that its postoperative course was comparable to that previously reported in the larger cohort (Schrader et al., 2026). Pairwise changes over time were analyzed using two-sided Wilcoxon signed-rank tests for paired samples. Comparisons were performed for the NCIQ total, TQ total, PSQ total, ADS-L, GAD-7, OI total, and speech intelligibility at 65 dB (FMT) across baseline, 6 months, 1 year, and 2 years after cochlear implantation. The six pairwise contrasts were baseline vs. 6 months, baseline vs. 1 year, baseline vs. 2 years, 6 months vs. 1 year, 6 months vs. 2 years, and 1 year vs. 2 years. Analyses used pairwise complete cases. To control for multiple testing, Holm-adjusted *p*-values were calculated separately within each parameter across the six contrasts. Effect sizes for Wilcoxon signed-rank tests were calculated as *r* = |Z|/sqrt(N), where N is the number of non-zero paired differences, and are reported in Supplementary Table S1. Statistical significance was set at *p* < 0.05.

In response to the reviewer’s request, an additional exploratory descriptive subgroup analysis of NCIQ total was conducted after stratification by baseline tinnitus burden (habituated tinnitus: TQ 0–46; unhabituated tinnitus: TQ ≥ 47). Due to small and uneven subgroup sizes, these analyses were only interpreted descriptively and are included in the Supplementary material.

## 3 Results

### 3.1 Participants, analytic cohort, and baseline characteristics

**Figure 1:**
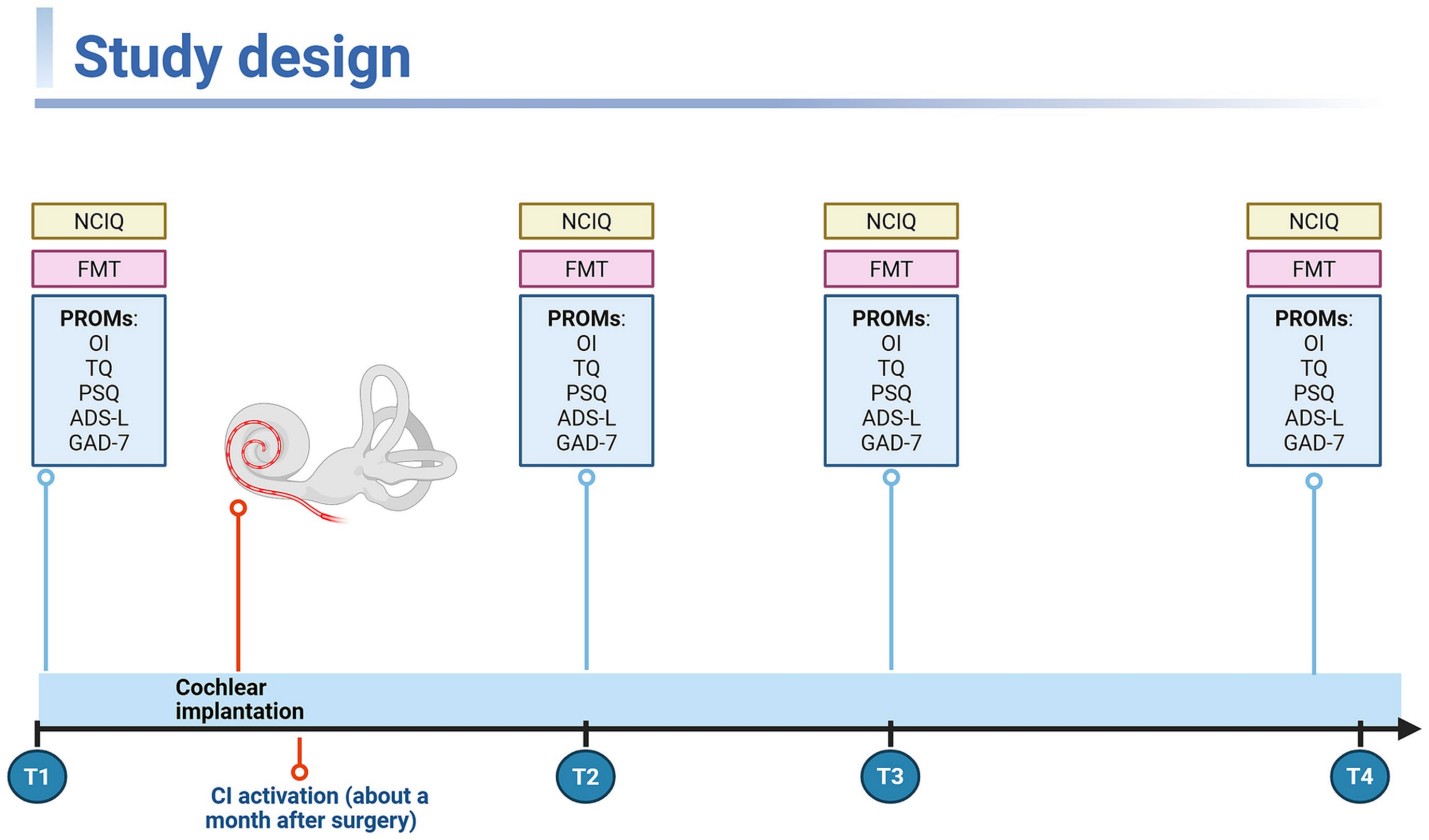
Study design and assessment schedule. Participants diagnosed with SSD and undergoing unilateral cochlear implantation were assessed at baseline (T1; pre-implantation) and at 6 months (T2), 1 year (T3), and 2 years (T4) post-implantation. At each time point, outcomes included the Nijmegen Cochlear Implant Questionnaire (NCIQ; total and subscales) and a battery of patient-reported outcome measures (PROMs) comprising the Oldenburg Inventory (OI), Tinnitus Questionnaire (TQ), Perceived Stress Questionnaire (PSQ), General Depression Scale (ADS-L), and Generalized Anxiety Disorder scale (GAD-7). Speech recognition was assessed using the Freiburg monosyllable test (FMT) in a free-field setup (0° azimuth) with the contralateral ear masked during measurement. Created in BioRender. Szczepek, A. (2026). https://BioRender.com/xl61plb.

Of the previously described longitudinal SSD cohort (*n* = 70), 36 patients (51.4%) had complete NCIQ data at baseline and at 6 months, 1 year, and 2 years after cochlear implantation and were therefore included in this secondary complete-case analysis, whereas 34 patients (48.6%) were not included because at least one required NCIQ assessment was missing. The study design and assessment schedule are shown in Figure 1. Baseline demographic, clinical, and patient-reported outcome characteristics of the analytic cohort are summarized in Table 1. In line with the study aims, the primary analyses focused on timepoint-specific cross-sectional associations between NCIQ total and the other PROMs, as well as on prospective rank-based regression models examining associations with 2-year NCIQ outcome; longitudinal pairwise comparisons were retained as a secondary descriptive analysis to characterize the complete-case subset.

**Table 1:** Baseline characteristics of the study cohort.

Characteristic	*n* (%)	Mean (SD)	Median [Q1–Q3]
Sample size	36		
Sex	Female: 23 (63.9%); male: 13 (36.1%)		
Implanted side	Left: 20 (55.6%); right: 16 (44.4%)		
Age at implantation (years)		57.17 (14.03)	56.00 [50.00–69.50]
NCIQ total		61.63 (12.77)	63.62 [50.68–69.87]
OI total		2.90 (0.43)	2.87 [2.67–3.27]
TQ total		29.0 (23.7)	24.0 [8.5–48.0]
PSQ total		0.42 (0.19)	0.38 [0.29–0.53]
ADS-L		17.33 (10.34)	14.00 [9.75–23.50]
GAD-7		6.14 (4.38)	5.50 [3.00–8.00]

Data are presented as *n* (%) for categorical variables and as mean (SD) or median [Q1–Q3] for continuous variables, as appropriate. SSD, single-sided deafness; PTA, pure-tone average; CI, cochlear implantation; TQ, tinnitus questionnaire; OI, Oldenburg inventory; PSQ, perceived stress questionnaire; ADS-L, general depression scale; GAD-7, general anxiety disorder 7-item scale; NCIQ, Nijmegen cochlear implant questionnaire.

### 3.2 Comparison of included and not-included patients

To assess potential selection effects related to the complete-case design, baseline characteristics of the 36 included patients were compared with those of the 34 patients from the parent cohort who were not included in the present analysis (Table 2). The two groups were broadly similar with respect to demographic variables and most baseline clinical measures. However, the included group showed significantly higher baseline perceived stress, along with trend-level differences toward poorer baseline subjective hearing and higher baseline anxiety (Table 2). Overall, these findings suggest that the complete-case cohort was broadly comparable to the remainder of the parent cohort, although it may have carried somewhat greater baseline psychosocial burden.

**Table 2:** Baseline comparison of included and not-included patients from the parent cohort.

Variable	Included (*n* = 36)	Not included (*n* = 34)	Statistical test	*p*-value
Demographic and clinical characteristics				
Age at implantation, years	57.17 ± 14.03 (*n* = 36)	60.45 ± 12.44 (n = 33)	Welch *t*-test	0.306
Duration of deafness, years	12.83 ± 17.86 (*n* = 30)	13.50 ± 19.43 (n = 16)	Welch *t*-test	0.910
Sex, male	13 (36.1%)	15 (44.1%)	Chi-square	0.660
Sex, female	23 (63.9%)	19 (55.9%)	Chi-square	0.660
Implanted side, left	20 (55.6%)	20 (58.8%)	Chi-square	0.534
Implanted side, right	16 (44.4%)	13 (38.2%)	Chi-square	0.534
Implanted side, missing/other	0 (0.0%)	1 (2.9%)	Chi-square	0.534
Baseline patient-reported outcome measures				
NCIQ total score	61.63 ± 12.77 (*n* = 36)	66.33 ± 14.34 (*n* = 28)	Welch *t*-test	0.179
OI total score	2.90 ± 0.43 (*n* = 36)	3.25 ± 0.96 (*n* = 28)	Welch *t*-test	0.083
TQ total score	29.00 ± 23.71 (*n* = 36)	30.28 ± 21.58 (*n* = 25)	Welch *t*-test	0.828
PSQ total score	0.42 ± 0.19 (*n* = 36)	0.30 ± 0.22 (*n* = 28)	Welch *t*-test	**0.025**
ADS-L score	17.33 ± 10.34 (*n* = 36)	13.32 ± 12.18 (*n* = 28)	Welch *t*-test	0.169
GAD-7 score	6.14 ± 4.38 (n = 36)	4.15 ± 3.95 (*n* = 27)	Welch *t*-test	0.064

### 3.3 Primary analysis: timepoint-specific associations between NCIQ and patient-reported outcome measures

Timepoint-specific Spearman correlations between NCIQ total and the other PROMs are presented in Table 3. Across all four assessment points, higher NCIQ total scores were consistently associated with lower tinnitus burden and better subjective hearing. Associations with depressive symptoms and anxiety were also observed throughout, whereas associations with perceived stress were not evident at baseline but emerged postoperatively and became stronger over time (Table 3). Overall, this correlation pattern indicates that disease-specific quality of life after cochlear implantation in SSD was related not only to subjective hearing performance, but also to tinnitus-related and broader psychosocial burden. The strongest association observed was the positive relationship between NCIQ total and OI total at 2 years (Table 3).

**Table 3:** Timepoint-specific spearman correlations between NCIQ total and patient-reported outcome measures.

Variable	Baseline rho	Baseline *p*-value	6 months rho	6 months *p*-value	1 year rho	1 year *p*-value	2 years rho	2 years *p*-value
TQ total	−0.539	<0.001	−0.449	0.007	−0.557	<0.001	−0.578	<0.001
OI total	0.650	<0.001	0.584	<0.001	0.665	<0.001	0.724	<0.001
PSQ total	−0.245	0.149	−0.414	0.013	−0.551	<0.001	−0.656	<0.001
ADS-L	−0.321	0.056	−0.419	0.012	−0.598	<0.001	−0.689	<0.001
GAD-7	−0.329	0.050	−0.408	0.015	−0.582	<0.001	−0.606	<0.001

### 3.4 Primary analysis: baseline and 6-month variables associated with 2-year NCIQ

Prospective rank-based regression models using baseline and 6-month PROMs to examine associations with 2-year NCIQ are summarized in Table 4. Both models were statistically significant, with the 6-month model explaining a larger proportion of variance than the baseline model (Table 4). In the baseline model, higher tinnitus burden was independently associated with lower 2-year NCIQ total score, whereas better subjective hearing and lower depressive symptom burden showed trend-level associations. In the 6-month model, higher tinnitus burden was again independently associated with lower 2-year NCIQ total, whereas better subjective hearing was independently associated with higher 2-year NCIQ total. Perceived stress, depressive symptoms, and anxiety were not independently associated with later NCIQ outcome when considered jointly in either model. Taken together, these findings identify tinnitus burden as the most consistent adverse factor and suggest that early postoperative subjective hearing status may be a particularly informative candidate early marker of later disease-specific quality-of-life outcome.

**Table 4:** Rank-based multiple regression models examining associations with 2-year total NCIQ score.

Predictor	B	*β*	95% CI for B	*p*
Baseline model (n = 36)				
OI total (baseline)	0.287	0.287	−0.002 to 0.576	0.051
TQ total (baseline)	−0.314	−0.314	−0.614 to −0.013	0.041
PSQ total (baseline)	−0.113	−0.113	−0.555 to 0.329	0.606
ADS-L (baseline)	−0.474	−0.473	−0.974 to 0.026	0.062
GAD-7 (baseline)	0.283	0.282	−0.187 to 0.753	0.228
**Model summary**	R = 0.675; R2 = 0.455; adjusted R2 = 0.364; F(5,30) = 5.009			0.002
6-month model (n = 35)				
OI total (6 months)	0.607	0.614	0.266 to 0.948	0.001
TQ total (6 months)	−0.363	−0.374	−0.651 to −0.075	0.015
PSQ total (6 months)	0.046	0.047	−0.413 to 0.504	0.840
ADS-L (6 months)	0.243	0.242	−0.281 to 0.766	0.351
GAD-7 (6 months)	−0.170	−0.175	−0.630 to 0.289	0.454
Model summary	R = 0.743, R2 = 0.552; adjusted R2 = 0.475; F(5,29) = 7.146			<0.001

Rank-based multiple regression was performed by transforming the dependent variable and all predictors into ranks, then fitting a standard multiple linear regression model to the ranked data. A higher NCIQ total indicates better health-related quality of life.

### 3.5 Secondary descriptive analysis: longitudinal postoperative change in the complete-case cohort

Longitudinal pairwise comparisons for the complete-case cohort are shown in Table 5. Overall, the postoperative course was characterized by early improvement followed by relative stabilization, consistent with the pattern previously observed in the parent cohort. NCIQ total, subjective hearing performance, and Freiburg monosyllable test performance all improved significantly from baseline to postoperative follow-up, with no significant differences among later postoperative assessments after correction for multiple testing. Tinnitus burden decreased significantly 6 months after CI and remained at that level across subsequent follow-up visits. However, the decrease was not clinically significant, as it was less than 12 TQ points (Hall et al., 2018), which was likely due to a relatively low baseline. Perceived stress showed significant early reductions, whereas depressive symptoms and anxiety did not show significant longitudinal changes after correction. Thus, the complete-case subset demonstrated the same general pattern of early postoperative benefit and subsequent stabilization as the parent cohort, supporting its use for the primary association and exploratory analyses.

**Table 5:** Secondary descriptive longitudinal pairwise comparisons in the complete-case cohort.

Outcome	Comparison (time points)	*n*	Time point 1, median [IQR]	Time point 2, median [IQR]	Wilcoxon W	Median change (later − earlier)	Holm-adjusted *p*-value
NCIQ total	Baseline vs. 6 months	36	63.62 [50.68–69.87]	67.57 [59.41–76.44]	115.0	3.78	0.006
NCIQ total	Baseline vs. 1 year	36	63.62 [50.68–69.87]	69.59 [55.45–76.54]	146.0	5.78	0.011
NCIQ total	Baseline vs. 2 years	36	63.62 [50.68–69.87]	69.08 [57.75–78.86]	139.0	5.98	0.009
NCIQ total	6 months vs. 1 year	36	67.57 [59.41–76.44]	69.59 [55.45–76.54]	308.0	0.43	1.000
NCIQ total	6 months vs. 2 years	36	67.57 [59.41–76.44]	69.08 [57.75–78.86]	293.0	−0.42	1.000
NCIQ total	1 year vs. 2 years	36	69.59 [55.45–76.54]	69.08 [57.75–78.86]	292.0	−0.01	1.000
TQ total	Baseline vs. 6 months	36	24.00 [8.75–48.00]	13.00 [4.00–25.00]	59.5	−6.50	<0.001
TQ total	Baseline vs. 1 year	36	24.00 [8.75–48.00]	9.50 [2.75–19.00]	31.5	−8.00	<0.001
TQ total	Baseline vs. 2 years	36	24.00 [8.75–48.00]	11.00 [2.00–27.75]	86.0	−8.50	0.001
TQ total	6 months vs. 1 year	36	13.00 [4.00–25.00]	9.50 [2.75–19.00]	135.0	0.00	0.909
TQ total	6 months vs. 2 years	36	13.00 [4.00–25.00]	11.00 [2.00–27.75]	158.0	0.00	0.912
TQ total	1 year vs. 2 years	36	9.50 [2.75–19.00]	11.00 [2.00–27.75]	164.5	0.00	0.912
PSQ total	Baseline vs. 6 months	36	0.42 [0.31–0.57]	0.32 [0.22–0.42]	98.0	−0.07	0.002
PSQ total	Baseline vs. 1 year	36	0.42 [0.31–0.57]	0.34 [0.22–0.43]	142.5	−0.08	0.024
PSQ total	Baseline vs. 2 years	36	0.42 [0.31–0.57]	0.34 [0.19–0.47]	161.0	−0.07	0.078
PSQ total	6 months vs. 1 year	36	0.32 [0.22–0.42]	0.34 [0.22–0.43]	239.5	0.01	1.000
PSQ total	6 months vs. 2 years	36	0.32 [0.22–0.42]	0.34 [0.19–0.47]	294.0	−0.01	1.000
PSQ total	1 year vs. 2 years	36	0.34 [0.22–0.43]	0.34 [0.19–0.47]	284.0	0.00	1.000
ADS-L	Baseline vs. 6 months	35	15.50 [9.00–24.50]	11.00 [7.00–18.50]	161.5	−3.00	0.199
ADS-L	Baseline vs. 1 year	36	15.50 [9.00–24.50]	13.00 [6.75–22.25]	215.0	−1.00	0.504
ADS-L	Baseline vs. 2 years	36	15.50 [9.00–24.50]	11.50 [7.75–22.50]	223.0	−2.00	0.525
ADS-L	6 months vs. 1 year	35	11.00 [7.00–18.50]	13.00 [6.75–22.25]	201.5	1.00	1.000
ADS-L	6 months vs. 2 years	35	11.00 [7.00–18.50]	11.50 [7.75–22.50]	193.5	0.00	1.000
ADS-L	1 year vs. 2 years	36	13.00 [6.75–22.25]	11.50 [7.75–22.50]	254.5	0.00	1.000
GAD-7	Baseline vs. 6 months	36	6.00 [2.75–9.00]	4.00 [1.75–7.25]	97.0	−1.00	0.051
GAD-7	Baseline vs. 1 year	36	6.00 [2.75–9.00]	4.00 [1.00–7.25]	140.0	−1.00	0.097
GAD-7	Baseline vs. 2 years	34	6.00 [2.75–9.00]	4.50 [1.25–6.75]	142.5	−1.00	0.414
GAD-7	6 months vs. 1 year	36	4.00 [1.75–7.25]	4.00 [1.00–7.25]	186.0	0.00	1.000
GAD-7	6 months vs. 2 years	34	4.00 [1.75–7.25]	4.50 [1.25–6.75]	127.5	0.00	1.000
GAD-7	1 year vs. 2 years	34	4.00 [1.00–7.25]	4.50 [1.25–6.75]	158.5	0.00	1.000
OI total	Baseline vs. 6 months	36	2.83 [2.08–3.29]	3.17 [2.67–3.88]	80.0	0.33	<0.001
OI total	Baseline vs. 1 year	35	2.83 [2.08–3.29]	3.50 [2.98–3.87]	45.0	0.53	<0.001
OI total	Baseline vs. 2 years	36	2.83 [2.08–3.29]	3.50 [3.00–4.08]	63.0	0.54	<0.001
OI total	6 months vs. 1 year	35	3.17 [2.67–3.88]	3.50 [2.98–3.87]	231.0	0.12	0.506
OI total	6 months vs. 2 years	36	3.17 [2.67–3.88]	3.50 [3.00–4.08]	257.0	0.17	0.684
OI total	1 year vs. 2 years	35	3.50 [2.98–3.87]	3.50 [3.00–4.08]	243.5	0.00	0.930
FMT at 65 dB, %	Baseline vs. 6 months	28	0.00 [0.00–0.00]	62.50 [44.38–75.00]	0.0	60.00	<0.001
FMT at 65 dB, %	Baseline vs. 1 year	28	0.00 [0.00–0.00]	67.50 [44.38–75.00]	0.0	65.00	<0.001
FMT at 65 dB, %	Baseline vs. 2 years	22	0.00 [0.00–0.00]	65.00 [50.00–73.75]	0.0	62.50	<0.001
FMT at 65 dB, %	6 months vs. 1 year	28	62.50 [44.38–75.00]	67.50 [44.38–75.00]	107.0	2.50	0.338
FMT at 65 dB, %	6 months vs. 2 years	22	62.50 [43.12–70.00]	65.00 [51.25–75.00]	61.0	2.50	0.338
FMT at 65 dB, %	1 year vs. 2 years	21	70.00 [45.00–70.00]	65.00 [55.00–75.00]	38.5	5.00	0.214

Pairwise longitudinal comparisons for NCIQ total, TQ total, PSQ total, ADS-L total, GAD-7 total, OI total, and Freiburg Monosyllable Test (FMT) performance at 65 dB across baseline, 6 months, 1 year, and 2 years after cochlear implantation in the complete-case cohort. Six pairwise contrasts were examined for each variable: baseline versus 6 months, baseline versus 1 year, baseline versus 2 years, 6 months versus 1 year, 6 months versus 2 years, and 1 year versus 2 years. Values are based on two-sided Wilcoxon signed-rank tests for paired samples. Holm-adjusted p-values were calculated separately within each variable across the six contrasts. Because the available statistical output reported the Wilcoxon test statistic rather than standardized *Z* values, W is presented.

An additional exploratory descriptive subgroup analysis stratified by baseline tinnitus burden indicated that patients with habituated tinnitus (TQ 0–46; *n* = 26) had consistently higher NCIQ total scores across follow-up than those with unhabituated tinnitus (TQ ≥ 47; *n* = 10). In the habituated subgroup, NCIQ total improved significantly from baseline to 6 months, 1 year, and 2 years (Wilcoxon *p* = 0.003, 0.006, and 0.008, respectively; Friedman *χ*^2^(3) = 10.674, *p* = 0.014, Kendall’s W = 0.137), whereas no significant within-group change was observed in the unhabituated subgroup (Wilcoxon *p* = 0.114, 0.241, and 0.093, respectively; Friedman *χ*^2^(3) = 2.280, *p* = 0.516, Kendall’s W = 0.076). Owing to the small and imbalanced subgroup sizes, these findings are presented only descriptively (Supplementary Table S2).

## 4 Discussion

This study extends our previously published 2-year SSD cohort report by addressing a different, more specific question: not whether outcomes improved after cochlear implantation, but which patient-reported domains were most closely linked to disease-specific health-related quality of life across follow-up and which early PROM characteristics were associated with later 2-year NCIQ outcome. Three main findings emerged. First, the complete-case subset showed the same overall postoperative pattern as the parent cohort, namely early improvement followed by relative stabilization, supporting its use for the present secondary analyses. Second, across all assessment points, lower NCIQ total scores were consistently associated with higher tinnitus burden and poorer subjective hearing, with additional associations involving depressive symptoms, anxiety, and, postoperatively, perceived stress. Third, in the exploratory prospective models, tinnitus burden emerged as the most consistent adverse marker, whereas better subjective hearing at 6 months was independently associated with better 2-year NCIQ outcome. Taken together, these findings suggest that long-term patient-reported benefits after cochlear implantation in SSD are influenced by a complex interaction of auditory, tinnitus-related, and psychosocial factors, and that early postoperative patient-reported status may help identify patients at risk of less favorable later quality-of-life adaptation, although this needs to be confirmed in larger, independent groups.

The secondary longitudinal analyses are important mainly because they show that the analytic subset behaved similarly to the previously reported parent cohort, rather than because they form the main contribution of the present study. In this complete-case sample, NCIQ total, tinnitus burden, subjective hearing performance, and speech intelligibility improved significantly from baseline to postoperative follow-up, with the largest changes occurring early and no significant differences among later postoperative assessments. This pattern is clinically plausible and aligns with the idea that the first postoperative months are the key period for auditory adaptation and device acclimatization after cochlear implantation in SSD (Lindquist et al., 2023; Galvin et al., 2019). It also supports the view that the subsequent association and exploratory multivariable analyses were carried out in a subgroup whose overall postoperative course was not substantially different from that of the original cohort.

The timepoint-specific correlation analyses further clarify the multidimensional nature of disease-specific quality of life after cochlear implantation in SSD. Across all four assessments, higher NCIQ total scores were consistently linked to lower tinnitus burden and better subjective hearing performance, while associations with depressive symptoms and anxiety were present throughout, and associations with perceived stress became evident after implantation. This pattern is clinically meaningful. In SSD, treatment success is rarely captured by a single domain because the burdens that motivate implantation often extend beyond audibility itself to include tinnitus-related distress, listening effort, reduced confidence in complex acoustic environments, and broader psychosocial strain. The present findings therefore support the view that the NCIQ outcome in SSD should be interpreted as an integrative, patient-centered construct rather than a simple proxy for auditory performance. They also align with previous reports showing that cochlear implantation in SSD may improve not only hearing-related functioning but also tinnitus burden and broader quality-of-life measures (Daher et al., 2023; Cuda et al., 2025; Shapiro et al., 2025; Wendrich et al., 2024; Kitterick et al., 2015; Lindquist et al., 2023; Tolisano et al., 2023), while substantial interindividual variability remains.

The multivariable analyses provide a more specific clinical message. Although the baseline model showed that preoperative symptom burden was already linked to later 2-year NCIQ outcomes, the 6-month model was more informative: higher tinnitus burden continued to be independently associated with lower 2-year NCIQ total scores, while better subjective hearing at 6 months was independently linked to better 2-year NCIQ results. One plausible interpretation is that preoperative subjective hearing in SSD still reflects significant compensation by the normal-hearing ear, while the 6-month postoperative OI score may better represent the patient’s actual daily benefit following device fitting, early auditory adaptation, and acclimatization to binaural input. Tinnitus burden may have become the most consistent adverse factor because, in SSD, tinnitus often constitutes a core part of the preoperative symptom load and can continue to limit perceived quality of life even as objective hearing improves. Practically, the 6-month postoperative visit seems to be a key clinical time point. Patients who still report considerable tinnitus-related distress or limited subjective hearing benefit at that stage might need closer, multidimensional follow-up and more personalized rehabilitation. However, given the sample size and exploratory nature of the models, these findings should be viewed as identifying potential early markers rather than confirmed predictors or definitive intervention thresholds.

A refined interpretation of these findings is especially vital in SSD, as the factors influencing patient-perceived success differ in part from those in traditional bilateral cochlear implant cases. In bilateral deafness, improvements in speech communication may be the primary perceived benefit, whereas in SSD, the value of implantation often hinges on relief from tinnitus, better hearing in challenging listening environments, and reduced daily psychosocial burdens. The present results align with that clinical reality. They suggest that even when objective performance improves, ongoing tinnitus distress may still limit perceived quality of life, while subjective hearing benefits become particularly significant once the implant offers a noticeable advantage in daily life despite reliance on the normal-hearing ear. Thus, outcome evaluation in SSD should remain multidimensional and not be limited to speech scores alone.

### 4.1 Clinical implications

From a clinical perspective, the present findings support a multidimensional approach to outcome evaluation after cochlear implantation in adults with single-sided deafness (SSD). When the goal is to improve disease-specific health-related quality of life, treatment success should not be judged solely by objective speech perception or a single overall hearing metric. Instead, the results indicate that tinnitus burden, subjective hearing experience, and broader psychosocial factors all shape patients’ perceptions of postoperative benefit. In particular, the consistent association between higher tinnitus burden and lower NCIQ total score suggests that persistent tinnitus-related distress may continue to constrain quality of life even when auditory performance improves.

**Figure 2:**
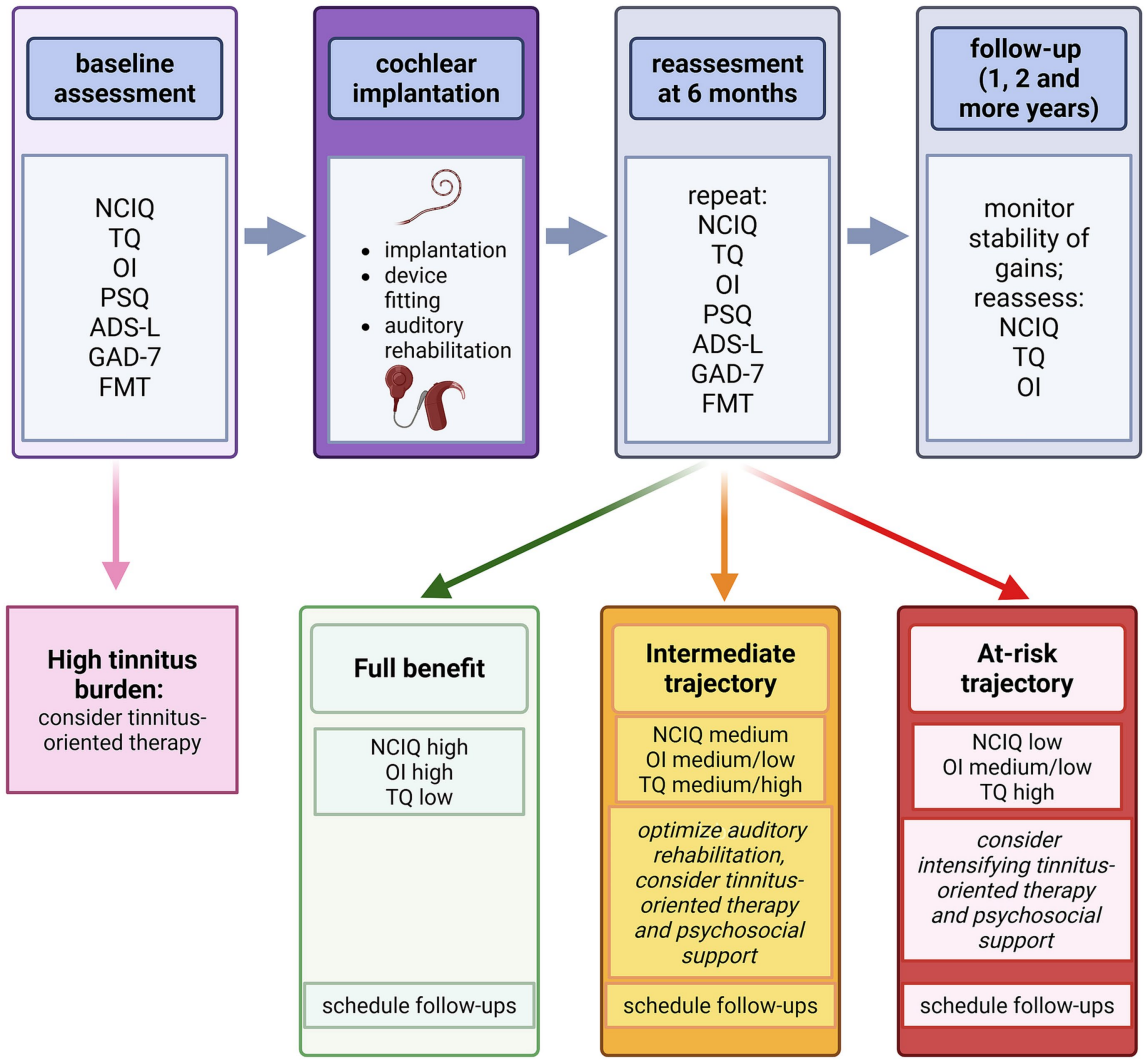
Conceptual model for PROM-informed postoperative follow-up. Conceptual interpretation of the present findings illustrating how multidimensional patient-reported follow-up may help identify patients at risk of less favorable long-term disease-specific quality-of-life outcomes after cochlear implantation in single-sided deafness. The framework emphasizes baseline and early postoperative assessments of health-related quality of life (NCIQ), tinnitus burden (TQ), subjective hearing performance (OI), psychological symptom burden [perceived stress (PSQ), depressive symptoms (ADS-L), and anxiety (GAD-7), and speech intelligibility (FMT)]. The 6-month postoperative assessment is highlighted as a clinically informative time point, as higher tinnitus burden and poorer subjective hearing at this stage were associated with less favorable 2-year NCIQ outcome. This figure is intended as a conceptual summary of the present results and not as a validated clinical decision algorithm. Created in BioRender. Szczepek, A. (2026). https://BioRender.com/xl61plb.

The exploratory multivariable analyses further suggest that the early postoperative period may be particularly important for clinical follow-up. While baseline symptom levels showed some association with later outcomes, the 6-month assessment appeared more clinically informative: patients with higher tinnitus severity and poorer subjective hearing at 6 months experienced less favorable 2-year disease-specific quality-of-life outcomes. This does not justify creating a formal predictive model or setting validated cutoff points based solely on the current sample, but it does highlight the practical value of structured PROM-based monitoring during the 6-month postoperative visit (Figure 2). Patients who continue to report significant tinnitus distress or limited subjective hearing improvement at that stage may benefit from closer follow-up and more personalized rehabilitative support. At the same time, the current data are not enough to recommend fixed triage thresholds or standardized treatment protocols, which should be developed in larger, confirmatory studies.

At the same time, the unexplained variance underscores the complex and multidimensional nature of long-term patient-reported outcomes after cochlear implantation in SSD. In addition to audiological status, central plasticity mechanisms, individual coping strategies, psychological comorbidities, duration of implant use, and rehabilitation intensity are all likely to influence later health-related quality of life (Häußler et al., 2020; Lindquist et al., 2023; Ketterer et al., 2020; Olze et al., 2022). This interpretation is consistent with international reports showing considerable interindividual variability in patient-reported outcomes after cochlear implantation in SSD (Arndt et al., 2011; Daher et al., 2023; Oh et al., 2023; Lindquist et al., 2023; Ketterer et al., 2020). The present analysis suggests that part of this variability can be attributed to tinnitus-related burden. Subjective hearing benefit appears to become more clinically informative after surgery, particularly once cochlear implantation provides a functional advantage that exceeds the preoperative compensatory capacity of the normal-hearing ear. However, if tinnitus-related distress persists, this benefit may still be insufficient to translate into a clearly improved quality of life.

These observations are particularly relevant in SSD, where the patient-perceived value of cochlear implantation often extends beyond speech understanding alone. For many patients, the overall benefit of implantation depends on the combined effects of tinnitus relief, improved hearing in everyday listening situations, reduced listening effort, and improved confidence and participation in daily life. Accordingly, routine postoperative assessment in SSD should remain broad and patient-centered. Disease-specific quality-of-life measures should be considered alongside tinnitus-, hearing-, and psychosocial PROMs, not as replacements for audiological testing but as complementary tools for identifying patients whose longer-term adaptation may require closer follow-up, more individualized counseling, intensified auditory rehabilitation, or additional psychosocial support. Only such a multidimensional approach allows the clinical relevance of these factors to be interpreted appropriately and translated into personalized postoperative care.

### 4.2 Limitations and outlook

Several limitations should be kept in mind when interpreting the results. First, this was a secondary complete-case analysis of a previously published prospective SSD cohort. Of the 70 patients in the original cohort, 36 (51.4%) had complete NCIQ data across all four required assessment points and were included, while 34 (48.6%) were excluded for missing at least one required NCIQ assessment. Although this design was suitable for the current association and exploratory multivariable questions, it may have introduced selection bias. In the baseline comparison, the included and excluded patients were mostly similar in most demographic and clinical variables, but the included group exhibited higher perceived stress and showed trend-level differences in subjective hearing and anxiety. The analytic subset may therefore represent a group that is somewhat more psychosocially burdened than the rest of the parent cohort, which could limit the extent to which the findings generalize. Detailed reasons for each missed follow-up assessment were not consistently available in this long-term real-world cohort, and no formal missing-data model or imputation analysis was conducted. Additionally, the increased psychological burden might have influenced the 36 patients’ willingness to remain in follow-up care. Furthermore, the study dataset lacked sufficiently standardized historical data on tinnitus onset, duration, prior tinnitus-specific treatments, or pre-existing psychiatric diagnoses, so these factors could not be analyzed. Second, the study was conducted at a single tertiary referral center, and the multivariable models were based on a small number of patients, which limits statistical power, constrains the number of variables that can be analyzed simultaneously, and increases the risk that the regression coefficients are specific to this sample. Accordingly, the rank-based regression results should be viewed as exploratory and hypothesis-generating, and the identified variables should be considered candidate early markers rather than validated predictors for individual-level prognostic use. Third, the current models primarily focus on PROMs and a single speech intelligibility measure. Other factors likely important for long-term CI outcomes in SSD, such as speech perception in noise, auditory spatial perception, duration and consistency of device use, rehabilitation intensity, coping style, and social support, were not available for inclusion and may explain part of the unexplained variance. Finally, the observational design does not allow for causal inference; the current data cannot determine whether tinnitus burden, subjective hearing experiences, and psychological distress operate independently, sequentially, or reciprocally over time.

These limitations also define the following steps in the research. Larger multicenter longitudinal studies should test whether early postoperative PROM profiles remain associated with later outcomes in larger cohorts across broader SSD populations, with external validation. Subsequent research should integrate PROMs with richer audiological and behavioral variables and examine whether postoperative changes in tinnitus burden or subjective hearing mediate longer-term quality-of-life adaptation. The clinical goal is not to replace audiological assessment with PROMs, but to determine how multidimensional follow-up may better identify patients who may benefit from more individualized rehabilitation and support after cochlear implantation in SSD.

### 4.3 Conclusion

In adults with single-sided deafness, disease-specific health-related quality of life after cochlear implantation appears to reflect a multidimensional process that extends beyond auditory rehabilitation alone. In this secondary complete-case analysis, higher tinnitus burden was consistently associated with poorer NCIQ outcome, whereas better subjective hearing at 6 months was associated with better 2-year disease-specific quality of life. These findings support structured, multidimensional postoperative follow-up in SSD and suggest that early postoperative patient-reported status may help identify patients who require closer monitoring or more individualized rehabilitation. At the same time, given the single-center design, modest sample size, complete-case approach, and exploratory nature of the multivariable analyses, these variables should be interpreted as candidate early markers that require confirmation in larger independent cohorts.

## Data Availability

The raw data supporting the conclusions of this article will be made available by the authors, without undue reservation.
