# Supplementary material for: Early tinnitus burden and subjective hearing are candidate markers of 2-year quality of life after cochlear implantation in single-sided deafness
Source: Front Neurosci. 2026 Apr 15;20:1832641. doi: 10.3389/fnins.2026.1832641 (PMC13124565; doi:10.3389/fnins.2026.1832641)
Supplement: Supplementary file 1 [file Table_1.DOCX]

**Supplementary Table S1.** Wilcoxon effect sizes for Table 5

| **Outcome** | **Comparison** | **n paired** | **N non-zero** | **Wilcoxon W** | **Z** | ***r*** |
| --- | --- | --- | --- | --- | --- | --- |
| NCIQ total | Baseline vs 6 months | 36 | 35 | 115.0 | -3.276 | 0.554 |
| NCIQ total | Baseline vs 1 year | 36 | 36 | 146.0 | -2.938 | 0.490 |
| NCIQ total | Baseline vs 2 years | 36 | 36 | 139.0 | -3.048 | 0.508 |
| NCIQ total | 6 months vs 1 year | 36 | 36 | 308.0 | -0.393 | 0.065 |
| NCIQ total | 6 months vs 2 years | 36 | 36 | 293.0 | -0.628 | 0.105 |
| NCIQ total | 1 year vs 2 years | 36 | 35 | 292.0 | -0.377 | 0.064 |
| TQ total | Baseline vs 6 months | 36 | 34 | 59.5 | -4.073 | 0.699 |
| TQ total | Baseline vs 1 year | 36 | 33 | 31.5 | -4.452 | 0.775 |
| TQ total | Baseline vs 2 years | 36 | 34 | 86.0 | -3.617 | 0.620 |
| TQ total | 6 months vs 1 year | 36 | 26 | 135.0 | -1.030 | 0.202 |
| TQ total | 6 months vs 2 years | 36 | 27 | 158.0 | -0.746 | 0.144 |
| TQ total | 1 year vs 2 years | 36 | 27 | 164.5 | -0.589 | 0.113 |
| PSQ total | Baseline vs 6 months | 36 | 36 | 98.0 | -3.582 | 0.597 |
| PSQ total | Baseline vs 1 year | 36 | 36 | 142.5 | -2.821 | 0.470 |
| PSQ total | Baseline vs 2 years | 36 | 36 | 161.0 | -2.405 | 0.401 |
| PSQ total | 6 months vs 1 year | 36 | 34 | 239.5 | -0.275 | 0.047 |
| PSQ total | 6 months vs 2 years | 36 | 34 | 294.0 | -0.130 | 0.022 |
| PSQ total | 1 year vs 2 years | 36 | 32 | 284.0 | -0.116 | 0.021 |
| ADS-L total | Baseline vs 6 months | 35 | 29 | 161.5 | -2.129 | 0.395 |
| ADS-L total | Baseline vs 1 year | 36 | 31 | 215.0 | -1.528 | 0.274 |
| ADS-L total | Baseline vs 2 years | 36 | 32 | 223.0 | -1.437 | 0.254 |
| ADS-L total | 6 months vs 1 year | 35 | 24 | 201.5 | -0.705 | 0.144 |
| ADS-L total | 6 months vs 2 years | 35 | 25 | 193.5 | -0.802 | 0.160 |
| ADS-L total | 1 year vs 2 years | 36 | 26 | 254.5 | -0.108 | 0.021 |
| GAD-7 total | Baseline vs 6 months | 36 | 30 | 97.0 | -2.607 | 0.476 |
| GAD-7 total | Baseline vs 1 year | 36 | 29 | 140.0 | -2.345 | 0.436 |
| GAD-7 total | Baseline vs 2 years | 34 | 24 | 142.5 | -1.700 | 0.347 |
| GAD-7 total | 6 months vs 1 year | 36 | 21 | 186.0 | -0.320 | 0.070 |
| GAD-7 total | 6 months vs 2 years | 34 | 15 | 127.5 | -0.153 | 0.039 |
| GAD-7 total | 1 year vs 2 years | 34 | 17 | 158.5 | -0.081 | 0.020 |
| OI total | Baseline vs 6 months | 36 | 33 | 80.0 | -4.179 | 0.727 |
| OI total | Baseline vs 1 year | 35 | 34 | 45.0 | -4.630 | 0.794 |
| OI total | Baseline vs 2 years | 36 | 35 | 63.0 | -4.488 | 0.759 |
| OI total | 6 months vs 1 year | 35 | 23 | 231.0 | -0.741 | 0.155 |
| OI total | 6 months vs 2 years | 36 | 29 | 257.0 | -0.550 | 0.102 |
| OI total | 1 year vs 2 years | 35 | 17 | 243.5 | -0.088 | 0.021 |
| FMT at 65 dB, % | Baseline vs 6 months | 28 | 28 | 0.0 | -4.629 | 0.875 |
| FMT at 65 dB, % | Baseline vs 1 year | 28 | 28 | 0.0 | -4.629 | 0.875 |
| FMT at 65 dB, % | Baseline vs 2 years | 22 | 22 | 0.0 | -4.110 | 0.876 |
| FMT at 65 dB, % | 6 months vs 1 year | 28 | 26 | 107.0 | -1.284 | 0.252 |
| FMT at 65 dB, % | 6 months vs 2 years | 22 | 18 | 61.0 | -1.339 | 0.316 |
| FMT at 65 dB, % | 1 year vs 2 years | 21 | 16 | 38.5 | -1.749 | 0.437 |

Effect sizes for all Wilcoxon signed-rank tests reported in Table 5. Supplementary Table S1 presents the Wilcoxon test statistic (W), standardized Z value, and effect size *r* = |Z| / √N for Wilcoxon signed-rank tests, where N is the number of non-zero paired differences. Interpretation: trivial < 0.10, small 0.10–0.29, medium 0.30–0.49, large ≥ 0.50.
