# Supplementary material for: Early tinnitus burden and subjective hearing are candidate markers of 2-year quality of life after cochlear implantation in single-sided deafness
Source: Front Neurosci. 2026 Apr 15;20:1832641. doi: 10.3389/fnins.2026.1832641 (PMC13124565; doi:10.3389/fnins.2026.1832641)
Supplement: Supplementary file 2 [file Table_2.DOCX]

**Supplementary Table 2**

**Wilcoxon signed-rank tests for within-group pairwise changes in NCIQ total (habituated vs unhabituated tinnitus)**

| **Comparison** | **Habituated: Z** | **Habituated: *p*** | **Unhabituated: Z** | **Unhabituated: *p*** |
| --- | --- | --- | --- | --- |
| Baseline vs 6 months | -2.946 | **0.003** | -1.580 | 0.114 |
| Baseline vs 1 year | -2.730 | **0.006** | -1.172 | 0.241 |
| Baseline vs 2 years | -2.654 | **0.008** | -1.682 | 0.093 |
| 6 months vs 1 year | -0.698 | 0.485 | -0.459 | 0.646 |
| 6 months vs 2 years | -0.622 | 0.534 | -0.459 | 0.646 |
| 1 year vs 2 years | -0.040 | 0.968 | -0.561 | 0.575 |

**Descriptive statistics for NCIQ total in the two tinnitus subgroups**

| **Subgroup** | **Time point** | **n** | **Mean** | **SD** | **Median [Q1–Q3]** |
| --- | --- | --- | --- | --- | --- |
| Habituated tinnitus (TQ 0-46) | Baseline | 26 | 66.43 | 10.40 | 67.46 [61.23–74.03] |
| Habituated tinnitus (TQ 0-46) | 6 months | 26 | 71.32 | 9.50 | 72.90 [65.57–77.64] |
| Habituated tinnitus (TQ 0-46) | 1 year | 26 | 72.14 | 11.36 | 74.80 [61.28–80.11] |
| Habituated tinnitus (TQ 0-46) | 2 years | 26 | 72.24 | 10.88 | 72.94 [65.75–79.29] |
| Unhabituated tinnitus (TQ ≥47) | Baseline | 10 | 49.15 | 9.70 | 47.33 [40.78–57.05] |
| Unhabituated tinnitus (TQ ≥47) | 6 months | 10 | 53.14 | 10.40 | 56.33 [41.83–60.94] |
| Unhabituated tinnitus (TQ ≥47) | 1 year | 10 | 52.15 | 12.93 | 51.19 [40.80–63.29] |
| Unhabituated tinnitus (TQ ≥47) | 2 years | 10 | 57.07 | 16.80 | 54.37 [46.16–63.22] |

**Overall, within-group longitudinal analyses**

| **Subgroup** | **n** | **Friedman χ²** | **df** | **p** | **Kendall's W** |
| --- | --- | --- | --- | --- | --- |
| Habituated tinnitus (TQ 0-46) | 26 | 10.674 | 3 | **0.014** | 0.137 |
| Unhabituated tinnitus (TQ ≥47) | 10 | 2.280 | 3 | 0.516 | 0.076 |
